# Enhanced Expression of Thaumatin-like Protein Gene (*LeTLP1*) Endows Resistance to *Trichoderma atroviride* in *Lentinula edodes*

**DOI:** 10.3390/life11080863

**Published:** 2021-08-23

**Authors:** Xiaolong Ma, Xiaolin Fan, Gangzheng Wang, Ruiping Xu, Lianlian Yan, Yan Zhou, Yuhua Gong, Yang Xiao, Yinbing Bian

**Affiliations:** 1 Institute of Applied Mycology, College of Plant Science and Technology, Huazhong Agricultural University, Wuhan 430070, China; longxiaoma@126.com (X.M.); fxlhzau@163.com (X.F.); wgzhau@163.com (G.W.); mushroomhzau@163.com (R.X.); yylaputa@163.com (L.Y.); yanzhoufbw@mail.hzau.edu.cn (Y.Z.); gongyuhua@mail.hzau.edu.cn (Y.G.); xyfungi@163.com (Y.X.); 2 Key Laboratory of Agro-Microbial Resource and Development, Ministry of Agriculture, Wuhan 430070, China

**Keywords:** *Lentinula edodes*, *Trichoderma atroviride*, resistance mechanism, LeTLP1

## Abstract

*Lentinula edodes* (shiitake mushrooms) is heavily affected by the infection of *Trichoderma atroviride*, causing yield loss and decreases quality in shiitake mushrooms. The selection and breeding of fungal-resistant *L. edodes* species are an important approach to protecting *L. edodes* from *T. atroviride* infection. Herein, a highly resistant *L. edodes* strain (Y3334) and a susceptible strain (Y55) were obtained by using a resistance evaluation test. Transcriptome analyses and qRT-PCR detection showed that the expression level of *LeTLP1* (*LE01Gene05009*) was strongly induced in response to *T. atroviride* infection in the resistant Y3334. Then, *LeTLP1-*silenced and *LeTLP1*-overexpression transformants were obtained. Overexpression of *LeTLP1* resulted in resistance to *T. atroviride*. Compared with the parent strain Y3334, *LeTLP1-*silenced transformants had reduced resistance relative to *T. atroviride.* Additionally, the *Le*TLP1 protein (Y3334) exhibited significant antifungal activity against *T. atroviride*. These findings suggest that overexpression of *LeTLP1* is a major mechanism for the resistance of *L. edodes* to *T. atroviride.* The molecular basis provides a theoretical basis for the breeding of resistant *L. edodes* strains and can eventually contribute to the mushroom cultivation industry and human health.

## 1. Introduction

*Lentinula edodes* (also known as Xiang Gu or shiitake), a widely cultivated edible mushroom, is famous for its nutritional properties and pharmacological effects, such as its hypocholesterolemic, anti-inflammatory, antitumor, and neuroprotective effects [1,2,3,4,5,6,7,8]. The cultivation, production, and consumption of shiitake are important nowadays not only in Asian countries but also in western countries. The white-rot basidioycete *L. edodes* grows on the wood logs, and it is now cultivated on the sterilized sawdust-based substrates [1,9,10]. In practice, making use of sawdust-based cultivation as a replacement for natural logs has contributed to the expansion of the production and consumption of *L. edodes* [11]. However, the cultivation of *L. edodes* is severely affected by the infection of *Trichoderma* spp., which overgrows mushroom mycelia and kills them, resulting in a reduction in the mushroom yield [12,13]. As a result, the green mold disease induced by *Trichoderma* spp. on *L. edodes* severely limits the sustainable development of the mushroom industry.

The main Trichoderma species affecting *L. edodes* in China are *T. harzianum*, *T. atroviride*, *T. viride*, *T. longibrachiatum*, *T. polysporum*, *T. pleuroticola*, and *T. oblongisporum* [12,13,14,15]. Among them, *T. harzianum* is the most general and widespread pathogen in mushrooms [14,16,17,18]. The mycelia of *T. atroviride* possess a stronger capacity of overgrowth on *L. edodes*, and they attack the mycelium of *L. edodes* [12]. The other five species—namely, *T. viride*, *T. longibrachiatum*, *T. polysporum*, *T. pleuroticola*, and *T. oblongisporum*—are rare in shiitake logs [12,19,20]. Given the widespread growth of *T. atroviride*, it is crucial to protect *L. edodes* from being infected by *T. atroviride* in order to promote the yield and quality of *L. edodes*.

Fungicides such as carbendazim, thiabendazole, and benomyl have been widely applied for the control of the green mold disease caused by *Trichoderma* in mushrooms [21,22]. Additionally, prochloraz and metrafenone might also be recommended for the control of green mold disease in mushrooms [13]. However, the increasing fungicides resistance level in fungi and the occurrence of fungicide residues may cause a negative impact on the health of living organisms. This has triggered a strong interest in the development of alternative methods for fungal control [23,24]. Although several studies on the screening of resistant *L. edodes* strains have been conducted by many researchers [12,16,18,25], the effects of *Trichoderma* spp. on *L. edodes* are still not well documented. Previous studies revealed that laccase expression was increased in *L. edodes* when it interacted with *Trichoderma* spp. [26,27]. *L. edodes* secreted lignocellulose, which might play a key role in the resistance to *Trichoderma* [27]. In addition, other edible basidiomycete mushrooms produce defense proteins, known as ribotoxin-like proteins (e.g., ageritin), which inhibit the growth of *Trichoderma* through the damage to fungal ribosomes and the consequent inhibition of protein synthesis [28]. However, the molecular basis of the resistance of *L. edodes* to *T. atroviride* is largely unknown.

Herein, 56 strains of *L. edodes* were collected, and the resistance levels of these strains against *T. atroviride* were evaluated. Based on a systematic resistance evaluation test, two typical strains (the highly resistant strain Y3334 and the susceptible strain Y55) were obtained. Furthermore, the molecular basis of the resistance of *L. edodes* to *T. atroviride* is elaborated upon. This study provides a theoretical basis for the breeding of resistant *L. edodes* strains and will be beneficial to the promotion of the development of the mushroom cultivation industry.

## 2. Materials and Methods

### 2.1. L. edodes and T. atroviride Strains

The 56 strains of *L. edodes* (Table S1) were collected and identified by the Institute of Applied Mycology, Huazhong Agriculture University [29,30,31]. The *T. atroviride* strain 92-1 was isolated and identified from logs with green mold disease in Suizhou, Hubei [12,30].

### 2.2. Evaluation of the Resistance of L. edodes to T. atroviride

The confrontation assay method was used to test the resistance levels of the 56 *L. edodes* strains against *T. atroviride* with a dual culture of each *L. edodes* strain and the *T. atroviride* strain 92-1 on PDA plates. Mycelial plugs (8 mm in diameter) were obtained from the 7 day old colonies of *L. edodes* strains and were inoculated onto PDA at 1 cm from the edge of Petri plates that were 9 cm in diameter at 25 °C in a dark environment. Eight days later, mycelial plugs (8 mm in diameter) of *T. atroviride* strain 92-1 were inoculated onto the PDA on the opposite side (1 cm away from the plate’s edge). The confrontation conditions of *T. atroviride* strain 92-1 against the mycelial growth of *L. edodes* were observed. Additionally, the infection rates (L_T_:L_L_) were calculated as described by Wang et al. (2018) [32]. Each test was repeated thrice.

### 2.3. Responses of the Highly Resistant and Susceptible L. edodes Strains to Infection with T. atroviride

Highly resistant (Y3334) and the susceptible (Y55) *L. edodes* strains were chosen as representative strains. The confrontations of the highly resistant (Y3334) and the susceptible (Y55) *L. edodes* strains with *T. atroviride* (92-1) were observed at 24, 48, 72, 96, and 120 h post-infection (hpi), as well as 30 days post-infection (dpi). Furthermore, the changes in the mycelium of *L. edodes* that was treated with *T. atroviride* were observed via SEM (scanning electron microscopy) at 24 and 48 hpi.

### 2.4. Comparative Transcriptomics Analysis

**Figure 1:**
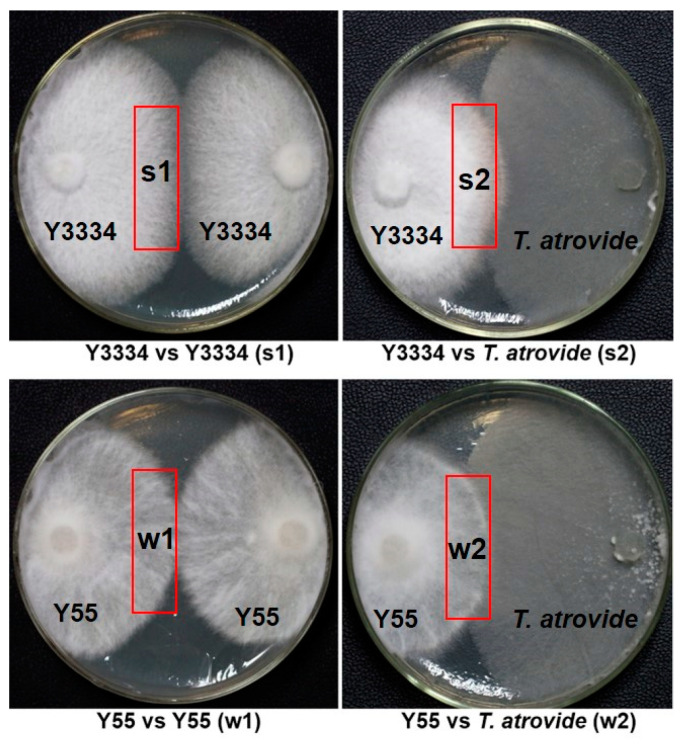
Collection of four mycelial samples (s1, s2, w1, and w2) of peripheral hyphal zones from *L. edodes* at the AC (after contact for 24 h) stage. The highly resistant (Y3334) and susceptible (Y55) *L. edodes* strains were grown on PDA plates that were covered with cellophane at 25 °C in a dark environment. The resistant and susceptible samples of the mycelia of *L. edodes* (s2, w2) were harvested after the *T. atrovide* strain 92-1 had made contact with the *L. edodes* strains for 24 h. As controls, the Y3334 strain and Y55 strain of *L. edodes* were confronted with themselves. The mycelial samples of resistant and susceptible *L. edodes* (s1 and w1) were harvested after contact for 24 h; s1 represents Y3334 vs. Y3334; s2 represents Y3334 vs. *T. atrovide* strain 92-1; w1 represents Y55 vs. Y55; w2 represents Y55 vs. *T. atrovide* strain 92-1. The red square represents the sampling location.

The highly resistant strain Y3334 and the susceptible strain Y55 were grown on PDA plates that were covered with cellophane at 25 °C in a dark environment. The mycelia of *L. edodes* were collected when the *T. atrovide* strain 92-1 had made contact with the *L. edodes* strain for 24 h. As controls, the Y3334 strain and Y55 strain of *L. edodes* were confronted with themselves. Four mycelia samples (s1, s2, w1, and w2) of the peripheral hyphal zones from *L. edodes* at the AC stage (after contact for 24 h) were collected (Figure 1) and immediately frozen in liquid nitrogen. There were three replicates of each sample. In total, twelve libraries that represented the highly resistant and susceptible *L. edodes* strains’ responses to *T. atrovide* or themselves were constructed for transcriptome sequencing (Illumina HiSeq).

#### 2.4.1. RNA Extraction, Library Preparation, and Sequencing

Mycelial samples were ground into fine powders in liquid nitrogen. Total RNAs were extracted using RNAiso Plus (TaKaRa, Dalian, China) and Fruit-mate^TM^ for the RNA Purification Kit (TaKaRa, Dalian, China) according to the manufacturer’s instructions. RNA concentrations and RNA integrity numbers were determined by using agarose gel electrophoresis and an Agilent 2100 Bioanalyzer (Agilent Technologies, Carpinteria, CA, USA), respectively. Amounts of 20 μg of RNA were equally isolated from mycelia for mRNA isolation, cDNA library construction, and sequencing according to the manufacturer’s instructions (Illumina, San Diego, CA, USA). The twelve libraries were sequenced through paired-end sequencing on an Illumina Hiseq 4000 sequencer (BGI). Sequencing services were provided by the Beijing Genomics Institute (BGI)-Shenzhen, Wuhan, China.

#### 2.4.2. Transcriptome Data Analysis

Twelve sets of raw data of all samples were pre-treated with FastQC, filtered, and trimmed via Trimmonmatic (parameter, PE and phred33). The indices were constructed via bowtie2 according to the *L. edodes* genome files (*L. edodes* W1-26 genome v1.0: http://shiitakegdb.chenlianfu.com/ accessed on 15 November 2019), followed by mapping of the RNA-seq reads to the reference genome of *L. edodes* with Tophat2 by using the default settings [33]. Subsequently, Samtools was used to create SAM files for the bam files [34]. Integrated and comprehensive transcript sets were obtained via Cufflinks and Cuffmerge. We ran the Cuffdiff function to find differentially expressed genes and transcripts in the samples using the default settings [33]. One-way ANOVA was applied, and a step-up FDR *p*-value was calculated to sort out the statistically significant differentially expressed genes (DEGs) by filtering the data at *p* < 0.05 (FDR) and with >2-fold change in the expression. The GO term enrichment and functional categorization for biological processes and cellular and molecular functions were carried out using Gene Ontology at the BGI databank.

### 2.5. qRT-PCR

The *L. edodes* mycelial samples for the RNA-seq analysis were also used for qRT-PCR. Gene-specific primers were designed using the Primer Premier 5.0 [35] for the target genes as well as the reference actin-1 gene (Table S2). The relative expression levels of the ten selected genes (Table S2) were calculated by using the 2^−ΔΔCT^ method [36].

### 2.6. Construction of LeTLP1-Overexpression and LeTLP1-Silencing Vectors and Fungal Transformation

The *LeTLP1-*overexpression vector was constructed by following the published protocols described by Yan et al. (2019) [37], with minor modifications. The CaMV 35S promoter of pCAMBIA1300 was replaced with the *Legpd* (*L. edodes* glyceraldehyde-3-phosphate dehydrogenase) promoter in order to produce the pCAMBIA1300-g vector. The *Legpd* promoter sequence was PCR amplified from the DNA of *L. edodes* strain W1-26 with the O-Legpd Promotor-F and O-Legpd Promotor-R primers (Table 1). The full length of the *LeTLP1* gene was amplified from *L. edodes* strain Y3334 cDNA with the O-LeTLP1-F and O-LeTLP1-R primers (Table 1), which contained the homologous arms, and then ligated into the pCAMBIA1300-g vector digested with *Eco*RI and *Kpn*I in order to generate the overexpression vector pCAMBIA1300-o-*LeTLP*. All constructs were assessed by sequencing analysis and transferred into *L. edodes* strain Y55 through *A. tumefaciens* EHA105 infection.

For the construction of the *LeTLP1*-silencing vector, a 500-bp antisense fragment from *LeTLP1* was amplified with the R-*LeTLP1*-F and R-*LeTLP1*-R primers (Table 1) from the cDNA of *L. edodes*. The *Leactin* promoter fragment was amplified with the R-*Leactin*-F and R-*Leactin*-R primers (Table 1) from the cDNA of *L. edodes*. Then, the *Leactin* promoter fragment, *LeTLP1* antisense fragment, and the aforesaid digested pCAMBIA1300-g vector were ligated in order to generate the silencing vector pCAMBIA1300-Ri-*LeTLP.* All constructs were assessed through sequencing analysis and transferred into *L. edodes* strain Y3334 through *A. tumefaciens* EHA105 infection.

The ten transformants were selected randomly to analyze their over-expression and silencing efficiency with qRT-PCR by using the primers for *LeTLP1* (*LE01Gene05009*) and by normalizing to the actin-1 gene by using the primers for the actin-1 gene (Table S2). The relative expression levels were calculated with the 2^−ΔΔCT^ method [36]. Three replicates for each isolate were analyzed, and the experiment was independently repeated three times.

### 2.7. Evaluation of the Level of Resistance against T. atroviride in LeTLP1-Overexpression and LeTLP1-Silenced Transformants

In order to test the effects of *LeTLP1* over-expression and silencing on the resistance of *L. edodes* against *T. atroviride*, the confrontation assays were undertaken according to the method presented in Section 2.2. The confrontations of the *LeTLP1* overexpression transformants (Y55_OE_3 and Y55_OE_10), silencing transformants (*TLP1*-Ri-3 and *TLP1*-Ri-8), and their parent isolates against *T. atroviride* (92-1) were observed on the 15th day after inoculation with *T. atroviride* in order to evaluate the level of the resistance of the transformants against *T. atroviride*, with five replicates for each group. Infection rates (L_T_:L_L_) were also calculated in order to evaluate the effects of *T. atroviride* on the mycelia of *L. edodes* [32].

### 2.8. Prokaryotic Expression of LeTLP1 and LeTLP (Y55)

For prokaryotic expression, the fragments (Figure S2) of *LeTLP1 and LeTLP* (Y55) cloned from Y3334 and Y55 fused with a 6 × His-tag were ligated into the plasmid pET32a (Novagen, WI, USA). The pET32a plasmids harboring *LeTLP1* and *LeTLP* (Y55) were transferred into the *E. coli* BL21(DE3) strain (Tiangen Biotech, Beijing, China). Then, each colony was induced in fresh LB broth with 40 μM isopropyl β-D-1-thiogalactopyranoside (IPTG). For purification of recombinant proteins, cultures were harvested after 16 h of induction at 18 °C. Suspensions were disrupted by squeezing in lysis buffer (20 mM Tris-HCl, 200 mM NaCl, 1 mM EDTA, pH 7.4). After centrifugation (7000× *g*, 4 °C, 10 min), the recombinant proteins in the *E. coli* lysate supernatant were purified using a nickel-affinity chromatography column (Yeasen, Shanghai, China) with an imidazole gradient elution. Recombinant proteins were verified with sodium dodecyl sulfate polyacrylamide gel electrophoresis (SDS-PAGE).

### 2.9. Assays of the Antifungal Activity of LeTLP1

The antifungal activity of *Le*TLP1 was measured by using the paper disc method described by Imtiaj and Lee (2007) [38]. Agar discs taken from 10 day old cultures of *T. atroviride* strain 92-1 were placed in the center of the Petri plates. The paper discs of Tris-HCl (pH 8.0), sterilized water, 20 μg of *Le*TLP (Y55), 5 μg of *Le*TLP1, 10 μg of *Le*TLP1, and 20 μg of *Le*TLP1 were separately placed at 1 cm from the edge of the Petri plates. As a control, agar discs of *T. atroviride* strain 92-1 were placed in the same manner on a fresh PDA plate with the paper discs of Tris-HCl (pH 8.0) and sterilized water. The inhibition rates (the percent inhibition of mycelial growth—PIMG) were calculated as described in Imtiaj and Lee (2007) [38]. The experiments were independently repeated three times.

### 2.10. Statistical Analysis

Following a one-way analysis of variance (ANOVA), Duncan’s multiple range test was used to evaluate significant differences in the gene expression levels, infection rates (L_T_:L_L_), and inhibition rates (the percent inhibition of mycelial growth—PIMG).

## 3. Results

### 3.1. Evaluation of Lentinula edodes Strains That Were Resistant to Trichoderma atrovide

The resistance levels of the 56 strains (Table S1) of *L. edodes* against *T. atroviride* were evaluated by using the resistance evaluation test (Table 2). The results revealed that the 56 *L. edodes* strains (21 cultivated strains and 35 wild strains) could be divided into a highly resistant group (Y3334, Y29, Y1515, Y121, and c67), medium-resistant group (++ labeled strains in Table 2), and susceptible group (Y55). Among them, the highly resistant strain Y3334 and the susceptible strain Y55 were chosen to be representative for the subsequent research on the resistance mechanism of *L. edodes* against *T. atroviride*.

### 3.2. Significant Differences in the Responses of the Highly Resistant and Susceptible L. edodes strains to T. atroviride Infection

**Figure 2:**
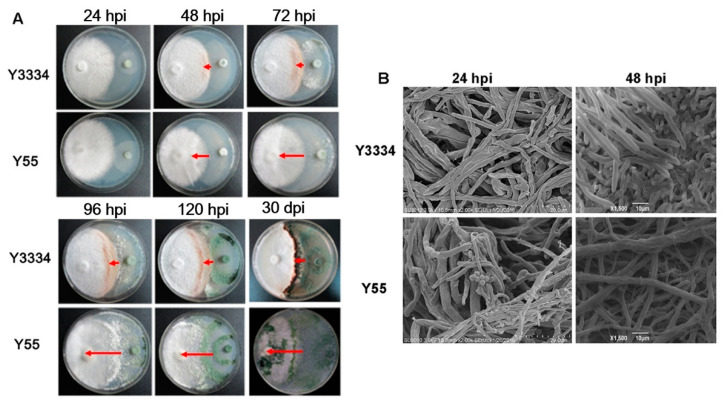
Photographs and SEM observations of the responses of highly resistant and susceptible *L. edodes* to *T. atroviride* inoculation. (**A**) The confrontations of the *L. edodes* Y3334 and Y55 strains with *T. atroviride* strain 92-1. The red arrows represent L_T_ (the length of the mycelia of *T. atroviride* covering *L. edodes*), as described by Wang et al. (2018) [32]. (**B**) SEM observations of the interactions of the *L. edodes* Y3334 and Y55 strains with *T. atroviride* strain 92-1 at 24 and 48 hpi, respectively. Bars = 10.0 μm.

On the PDA plate, significant differences were shown in the interactions of the highly resistant strain Y3334 and susceptible strain Y55 with *T. atroviride* at 24, 48, 72, 96, and 120 h post-infection (hpi) and 30 days post-infection (dpi), respectively (Figure 2A). Notably, the infection rates (L_T_:L_L_) were significantly lower in the confrontation of the highly resistant strain Y3334 with *T. atroviride* than for the susceptible strain Y55 at 24, 48, 72, 96, and 120 h, as well as 30 d (Figure 2A). Furthermore, we observed that the mycelia of *T. atroviride* became ruptured and rough, and conidia were not generated in the zone of interaction between the hyphae of *L. edodes* (Y3334) and *T. atroviride*. By contrast, the mycelium of *T. atroviride* was smooth and straight, and many conidia that adhered to the surface of the susceptible *L. edodes* strain Y55 were generated (Figure 2B).

### 3.3. Transcriptional Responses of the Highly Resistant and Susceptible L. edodes strains to T. atroviride

In order to screen the candidate genes for conferring resistance to *T. atroviride* in *L. edodes*, we synthesized twelve cDNA libraries from the mycelia of *L. edodes*, which were collected from the peripheral hyphal zones of Y3334 vs. *T. atroviride* (s2), Y55 vs. *T. atroviride* (w2), Y3334 vs. Y3334 (s1), and Y55 vs. Y55 (w1) (Figure 1). This was the first time that transcriptome RNA-sequencing data were generated for the Y3334 and Y55 strains of *L. edodes*.

**Figure 3:**
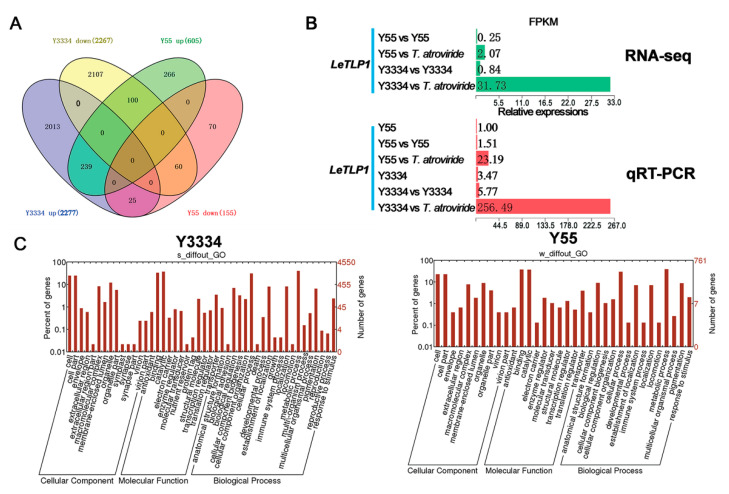
Transcriptional responses of the highly resistant and susceptible *L. edodes* strains to *T. atroviride.* (**A**) Venn diagram of upregulated and downregulated genes in *L. edodes*. (**B**) Significantly up-regulated *LeTLP1* in the Y3334 and Y55 strains. The mycelial samples (s1, s2, w1, and w2) of the peripheral hyphal zones were harvested from *L. edodes* at the AC (after contact for 24 h) stage. s1: Y3334 vs. Y3334; s2: Y3334 vs. *T. atrovide* strain 92-1; w1: Y55 vs. Y55; w2: Y55 vs. *T. atrovide* strain 92-1. (**C**) The GO terms of differentially expressed genes in the Y3334 and Y55 strains.

The DEGs were obtained by comparing the gene expressions among the s2, w2, s1, and w1 groups. As shown in the Venn diagram, a total of 4880 DEGs were obtained. In both the Y3334 and Y55 strains, 239 up-regulated genes were identified (Figure 3A). Among them, ten genes (Table S2) were selected to validate our differentially expressed gene results using qRT-PCR. The qRT-PCR results were correlated with the RNA-seq data (R^2^ = 0.8575, Figure S1), which confirmed the high reliability of the RNA-seq data in this study. Interestingly, the expression level of the thaumatin-like protein gene *LeTLP1* (*LE01Gene05009*) was strikingly increased in the resistant Y3334 when responding to infection with *T. atroviride*, and the *LeTLP1* expression level was extremely significantly higher than that in the susceptible Y55 in response to *T. atroviride* infection (Figure 3B).

The Gene Ontology enrichment analysis demonstrated that the most dominant groups among the biological process components were metabolic processes and cellular processes in both the Y3334 and Y55 strains (Figure 3C). The growth and death GO terms were only found in the highly resistant strain Y3334, while they were not found in the susceptible strain Y55. The genes in the growth and death GO terms might be resistance-related candidate genes. Meanwhile, the catalytic and binding activities were the most abundant functional groups among the molecular functions, implying that enzyme encoding genes (such as *LeTLP1*) may play key roles in the regulation of the resistance of *L. edodes to T. atroviride.*

### 3.4. Molecular Characterization of LeTLP1

**Figure 4:**
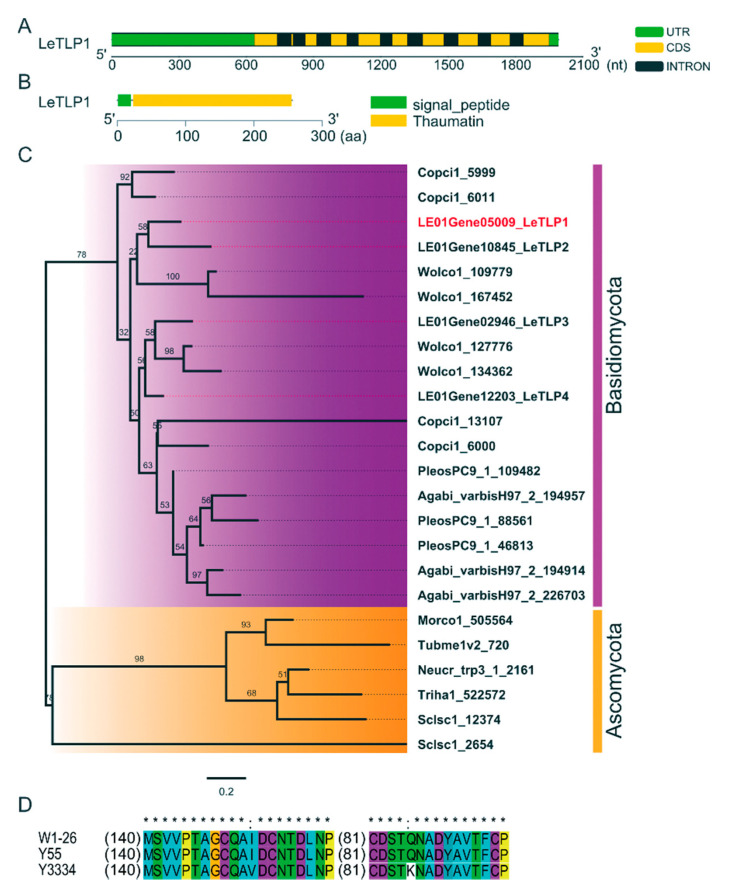
Molecular characterization of *LeTLP1*. (**A**) Structure of *LeTLP1*. (**B**) The prediction of the conserved domain in *Le*TLP1. (**C**) Molecular phylogenetic tree of *TLP* generated with the neighbor-joining (NJ) method using MEGA 7.0. One thousand bootstrap replicates were calculated, and the bootstrap values are shown at each node. Accession numbers for the sequences are listed on the clades of the phylogenetic tree. They are recorded in JGI (https://genome.jgi.doe.gov/portal/ accessed on 5 January 2020) as follows: LE01Gene (*L. edodes* W1-26 v1.0), Agabi_varbisH97_2 (*Agaricus bisporus* var bisporus(H97) v2.0), Copci1 (*Coprinopsis cinerea*), PleosPC9_1 (*Pleurotus ostreatus* PC9 v1.0), Wolco1 (Wolfiporia cocos MD-104 SS10 v1.0), Morco1 (*Morchella importuna* CCBAS932 v1.0), Neucr_trp3_1 (*Neurospora crassa* FGSC73 trp-3 v1.0), Sclsc1 (*Sclerotinia sclerotiorum* v1.0), Triha1 (*Trichoderma harzianum* CBS 226.95 v1.0), and Tubme1v2 (*Tuber melanosporum* Mel28 v1.2). (**D**) Multiple alignments of the amino acid sequences of *Le*TLP1 from W1-26, Y55, and Y3334. * represents the same amino acid residues at the site; represents the different amino acid residues at the site. () indicates that the amino acid sequences are the same in the region.

The full length of *LeTLP1* was 1993 bp. The DNA and cDNA sequence analyses revealed that the 768-bp ORF of *LeTLP1* was interrupted by nine introns (Figure 4A). The *LeTLP1* gene was predicted to encode a protein of 255 amino acids that contained a signal peptide and a conserved thaumatin domain (Accession pfam00314, E-Value 1.14894e-96), which belonged to the GH64-TLP-SF superfamily (Figure 4B). The phylogenetic analysis of the *TLP* indicated that *LeTLP1* was clustered with the *LeTLP2,* while it was separate from *LeTLP3*, *LeTLP4*, and the *TLP* genes of other fungi (Figure 4C). The amino acid sequences of *Le*TLP1 from W1-26 and Y55 were exactly the same, while the amino acid sequence of *Le*TLP1 from the resistant strain Y3334 exhibited two mutation sites (I-152-V and Q-245-K) compared to that of the sensitive strain Y55 (Figure 4D).

### 3.5. Effects of LeTLP1 Overexpression on the Resistance of L. edodes against T. atroviride

**Figure 5:**
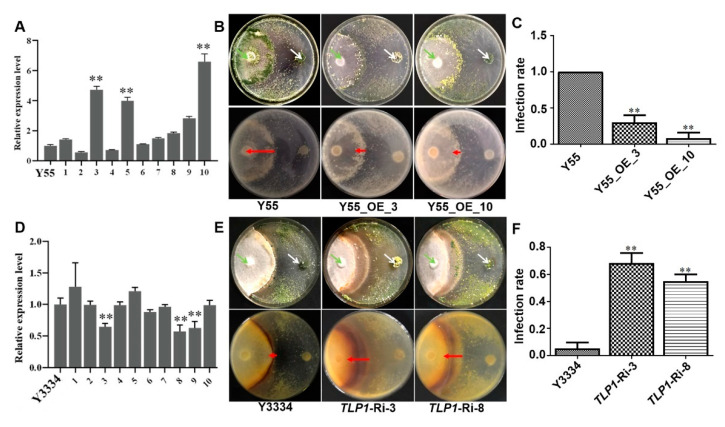
The effects of *LeTLP1* overexpression on the resistance of *L. edodes* to *T. atroviride* infection. (**A**) Relative expression levels of *LeTLP1* in the parent isolate Y55 of *L. edodes* and its *LeTLP1-*overexpression transformants; 1–10: Y55_OE_1, 2, 3, 4, 5, 6, 7, 8, 9, 10. (**B**) The confrontations of the *LeTLP1* overexpression transformants (Y55_OE_3 and Y55_OE_10) and the susceptible Y55 *L. edodes* against *T. atroviride* (92-1) were observed on the 15th day after inoculation with *T. atroviride*. (**C**) Infection rates (L_T_:L_L_) of *T. atroviride* in the susceptible Y55 strain and the *LeTLP1*-overexpression transformants. (**D**) Relative expression levels of *LeTLP1* in the parent isolate Y3334 of *L. edodes and its LeTLP1-*silenced transformants; 1–10: *TLP1*-Ri-1, 2, 3, 4, 5, 6, 7, 8, 9, 10. (**E**) The confrontations of the *LeTLP1*-silenced transformants (*TLP1*-Ri-3, *TLP1*-Ri-8, and *TLP1*-Ri-9) and the susceptible *L. edodes* (Y55) against *T. atroviride* (92-1) were observed on the 15th day after inoculation with *T. atroviride*, respectively. (**F**) Infection rates (L_T_:L_L_) of *T. atroviride* in the resistant strain Y3334 and *LeTLP1* RNAi transformants. Bars denote the standard error of the mean of three replicates in each of three independent experiments. ** indicates a statistically significant difference (*p* < 0.01). Green arrows refer to mycelial plugs of *L. edodes*; white arrows refer to mycelial plugs of *T. atroviride.* Red arrows represent L_T_ (the length of *T. atroviride* mycelia covering *L. edodes*), as described by Wang et al. (2018) [32].

Compared with the parent isolate Y55, the gene expression levels of *LeTLP1* in the three *LeTLP1-*overexpression transformants Y55_OE_3, Y55_OE_5, and Y55_OE_10 increased (*p* < 0.01) 4.5-fold, 4.0-fold, and 6.5-fold, respectively (Figure 5A). For two *LeTLP1*-overexpression strains (Y55_OE_3 and Y55_OE_10), significant differences in the responses of the *LeTLP1*-overexpression transformants and the susceptible (Y55) *L. edodes* strain to the infection with *T. atroviride* were observed (Figure 5B). The susceptible (Y55) *L. edodes* strain was completely covered by the mycelia and spores of *T. atroviride*, while only a few *T. atroviride* spores were observed on the surface of the mycelia of the *LeTLP1-*overexpression transformants. Notably, the infection rates (L_T_:L_L_) of *T. atroviride* in the mycelia of *LeTLP1*-overexpression transformants were significantly decreased compared to those in the parent isolate Y55 (Figure 5C). The results demonstrated that the overexpression of *LeTLP1* played a crucial role in the resistance of *L. edodes* to *T. atroviride.*

Compared with the parent isolate Y3334, the transcription levels of *LeTLP1* in the three silenced transformants *TLP1*-Ri-3, *TLP1*-Ri-8, and *TLP1*-Ri-9 declined by 35%, 43%, and 38%, respectively (Figure 5D). For two *LeTLP1* RNAi strains (*TLP1*-Ri-3 and *TLP1*-Ri-8), deep black compounds were generated in the interaction zone between the mycelia of the *LeTLP1* RNAi strains and the *T. atroviride* strains (Figure 5E), and the infection rates (L_T_:L_L_) of *T. atroviride* in the mycelia of *L. edodes* increased dramatically compared to those in the parent isolate Y3334 (Figure 5F). The results indicated that the resistance levels significantly declined when *LeTLP1* was silenced in *L. edodes*.

### 3.6. Antifungal Activity of LeTLP1 against T. atroviride

**Figure 6:**
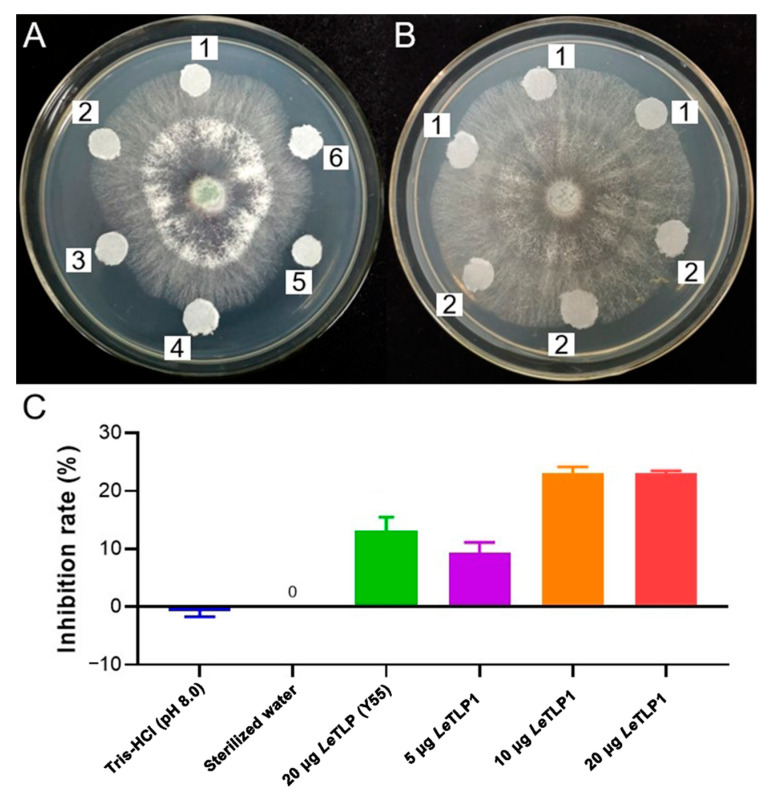
Antifungal activity of *Le*TLP1 against *T. atroviride*. (**A**,**B**) The antifungal activity of *Le*TLP1 was measured by using the paper disc method described by Imtiaj and Lee (2007) [38]. Agar discs taken from 10 day old cultures of *T. atroviride* strain 92-1 and were placed in the center of the Petri plates. 1–6: The paper discs of Tris-HCl (pH 8.0), sterilized water, 20 μg of *Le*TLP (Y55), 5 μg of *Le*TLP1, 10 μg of *Le*TLP1, and 20 μg of *Le*TLP1. (**C**) The inhibition rates (the percent inhibition of mycelial growth—PIMG) were calculated as described in Imtiaj and Lee (2007) [38].

The filter paper discs that contained the purified *Le*TLP1 obviously inhibited the mycelial growth of *T. atroviride* (Figure 6A,B). On the other hand, *Le*TLP1 was found to be more effective than *Le*TLP (Y55) against the mycelial growth of *T. atroviride* (Figure 6C). The results demonstrated that *Le*TLP1 exhibited significant antifungal activity against *T. atroviride*.

## 4. Discussion

The nutritional compounds and pharmacological properties of *Lentinula edodes* are beneficial to human health [1,2,3,4,5,6,7,8]. Unfortunately, *L. edodes* is heavily attacked by *Trichoderma* spp., which compete for limited resources and space for mycelial growth, and it harms the yield and quality of shiitake cultivation [12,16,17,39,40]. In order to protect *L. edodes* from the infection with *T. atroviride,* the screenings of resistant *L. edodes* germplasms and breeding of resistant *L. edodes* strains are eco-friendly and safe alternatives to the use of chemical fungicides [12,16,18]. Deciphering the molecular mechanisms of the resistance of *L. edodes* to *T. atroviride* will provide a theoretical basis for the breeding of resistant *L. edodes* strains.

In this study, significant differences in the responses of the highly resistant (Y3334) and susceptible (Y55) *L. edodes* strains to infection with *T. atroviride* were observed (Figure 2). A previous study demonstrated that *T. atroviride* possesses a strong capacity for attacking the mycelia of the susceptible *L. edodes* strain [12]. The SEM observations (Figure 2B) showed that the susceptible Y55 strain was heavily infected by *T. atroviride*, while the highly resistant Y3334 strain grew healthily. Therefore, the highly resistant Y3334 strain and the susceptible Y55 strain were proper materials for studying the molecular basis of the resistance of *L. edodes* to *T. atroviride*.

By using a transcriptomic analysis, we identified a novel candidate resistance-related gene, *LeTLP1* (*LE01Gene05009*), which contains a conserved thaumatin domain (Figure 3 and Figure 4B) and belongs to the group of thaumatin-like protein (TLPs). *LeTLP1* was strikingly significantly induced when the resistant *L. edodes* strain Y3334 responded to *T. atroviride* infection in comparison with the response of susceptible *L. edodes*, and this was verified by using qRT-PCR detection (Figure 3B). Similarly, thaumatin-like proteins (TLPs) were shown to be induced in rice plants that were infected with *Rhizoctonia solani* [41]. *Le*TLP1 (Figure S2) shares 16 conserved cysteines (Cys) that are required for eight disulfide bonds, as well as other TLPs, which play important roles in maintaining the structure and activity [42,43]. However, the functions of the novel *LeTLP1* in *L. edodes* and whether it regulates the resistance to *T. atroviride* are yet unknown.

In plants, TLPs are known as PR-5 proteins, and some of TLPs are known to exhibit both β-1,3-glucan binding [27,44,45] and endo-β-1,3-glucanase activities [46]. β-glucan, the most abundant fungal cell wall polysaccharide in fungi, serves in microbe-associated molecular patterns (MAMPs) [47]. In fungal cell walls, the most abundant β-glucan is β-1,3-glucan, which consists of dominant β-glucan content [48]. The TLPs crack the β-1,3-glucan in the cell walls of fungi and exhibit antifungal activity [27,46]. Furthermore, recent studies found that the TLPs also occurred in *L. edodes* [42,46], and they were highly conserved in plants exhibiting endo-β-1,3-glucanase activity. A previous study indicated that TLPs exhibit antifungal activity by lysing the β-1, 3-glucan in the cell walls of pathogenic fungi. TLPs may participate in regulating the level of resistance of *L. edodes* relative to invading fungi. In addition, the overexpression of the rice *TLP* gene in transgenic cassava resulted in enhanced tolerance to *Colletotrichum gloeosporioides* [49]. Thaumatin-like protein (Pe-TLP) acted as a positive factor in the enhanced resistance to spot disease in transgenic poplars [50]. Therefore, we asked whether the novel *LeTLP1* regulates the resistance level in *L. edodes*. Interestingly, the *LeTLP1-*overexpression experiment (Figure 5) demonstrated that the overexpression of *LeTLP1* contributes to the enhancement of the level of resistance of *L. edodes* against *T. atroviride.* The resistance levels of *LeTLP1-*silenced transformants (*TLP1*-Ri-3 and *TLP1*-Ri-8) declined. Our results demonstrated that the overexpression of *LeTLP1* plays a crucial role in the resistance of *L. edodes* to *T. atroviride.*

It is noteworthy that a thaumatin-like protein (TLG1) from *L. edodes* degrades lentinan (a β-1,3-glucan found in its own cell wall) and, thus, may play a role in cell wall degradation [42]. How does *L. edodes* protect itself against the activity of *Le*TLP1? Our results showed that the expression level of *LeTLP1* was very low without *T. atroviride* infection (Figure 3B). Therefore, the production of *Le*TLP1 is likely to be very limited in mycelia grown in a sawdust culture. This indicates that there is not enough to inflict any harm on itself. However, the *LeTLP1* gene expression level was strongly increased in the resistant *L. edodes* in response to the infection with *T. atroviride* (Figure 3B). *Le*TLP1 might affect the mycelia of *L. edodes* in response to the infection with *T. atroviride*. However, the SEM observations (Figure 2B) confirmed that the highly resistant *L. edodes* Y3334 strain grew healthily in a dual culture with *T. atroviride*. Consistently, *L. edodes tlg1* was not transcribed in vegetative mycelia grown in a liquid culture or sawdust culture, suggesting that it was induced under stress conditions [42]. Notably, a previous study indicated that the thaumatin-like protein (TLG1) might not act as an antifungal agent [42]. However, our results demonstrated that the *Le*TLP1 protein exhibited significant antifungal activity against *T. atroviride* (Figure 6). Taking the results together, this study demonstrates that the overexpression of *LeTLP1* in *L. edodes* results in resistance to *T. atroviride.*

## 5. Conclusions

We obtained a highly resistant *L. edodes* strain (Y3334) and a susceptible strain (Y55) and found a novel resistance-related gene, *LeTLP1 (LE01Gene05009)*, in the resistant *L. edodes* strain (Y3334). Further analysis showed that the *LeTLP1* gene expression level strongly increased in the resistant *L. edodes* in response to the infection with *T. atroviride* compared with the susceptible *L. edodes* strain. However, the expression level of *LeTLP1* was very low without the *T. atroviride* infection. More importantly, the experiment with *LeTLP1* overexpression and silencing clarified that the expression of *LeTLP1* was highly correlated with the level of resistance of *L. edodes* against *T. atroviride.* In addition, the *Le*TLP1 protein (Y3334) exhibited significant antifungal activity against *T. atroviride*. *Le*TLP1 was found to be more effective than *Le*TLP (Y55) against the mycelial growth of *T. atroviride*. This study demonstrates that resistance to *T. atroviride* is endowed by the enhanced expression of *LeTLP1* in *L. edodes*, thus providing insights into the molecular mechanisms of the resistance of *L. edodes* against *T. atroviride.* Further characterization of the upstream activators and downstream targets of *LeTLP1* will be used to decipher the resistance mechanisms in *L. edodes* and other basidiomycetes.

## Figures and Tables

**Table 1:** Sequence information of the primers used for the construction of *LeTLP1*-overexpression and *LeTLP1*-silencing vectors.

Primer Name	Primer Sequence (5′ to 3′)
O-Legpd Promotor-F	catATTCAAGCAGTCAATGGATTGGA
O-Legpd Promotor-R	tctagaggatccccgggtaccCGAAGTTTGAGGTGGTTGCG
O-LeTLP1-F	ccacctcaaacttcggaattcTCAGGGGCAAAATGTAACAGCATA
O-LeTLP1-R	ccattgactgcttgaatATGATGAAGAATTCTATCATTATCTCTGC
R-*LeTLP1*-F	agctcttcacgGATCCACTGCAGCAGTTGGG
R-*LeTLP1*-R	tgcttgaatTGGCCGGCAATCTTCACG
R-*Leactin*-F	ccacctcaaacttcggaattcGCAGTATTTATACCTACGGAGC
R-*Leactin*-R	cagtggatcCGTGAAGAGCTGCGAGTGTTG

**Table 2:** Evaluation of the resistance of *L. edodes* strains to *T. atrovide*.

Strains	AC about 7 Days	AC about 30 Days	Strains	AC about 7 Days	AC about 30 Days
c2	+	++	Y30	+	++
c6	+	++	Y37	+	++
c9	+	++	Y39	+	++
c10	+	++	Y51	+	++
c20	+	++	Y55	−	−
c23	+	++	Y70	+	++
c27	+	++	Y76	+	++
c28	+	++	Y78	+	++
c31	+	++	Y79	+	++
c42	+	++	Y84	+	++
c47	+	++	Y88	+	++
c48	+	++	Y89	+	++
c49	+	++	Y91	+	++
c50	+	++	Y94	+	++
c51	+	++	Y100	+	++
c64	+	++	Y104	+	++
c67	+	+++	Y110	+	++
c82	+	++	Y111	+	++
c85	+	++	Y113	+	++
c87	+	++	Y118	+	++
c88	+	++	Y121	+	+++
Y1	+	++	Y234	+	++
Y5	+	++	Y358	+	++
Y7	+	++	Y366	+	++
Y8	+	++	Y1515	+	+++
Y11	+	++	Y1518	+	++
Y14	+	++	Y3334	+	+++
Y29	+	+++	Y3353	+	++

AC means after contact; − represents susceptible; + represents the low resistance level; ++ represents the medium resistance level; +++ represents the high resistance level.

## Data Availability

The RNA-seq data generated for this study have been deposited into the Biotechnology Information (NCBI) Sequence Read Archive (SRA) under accession number: PRJNA590542.

## Supplementary Materials

The following are available online at https://www.mdpi.com/article/10.3390/life11080863/s1, Figure S1: qRT-PCR results of ten selected genes and the correlations between transcripotome data and real-time PCR results, Figure S2: Alignment of the amino acid sequences of *Le*TLP1 (Y3334), *Le*TLP (Y55), and *L. edodes* TLP (GenBank: AB244759), Table S1: Cultivated and wild strains of *Lentinula edodes* used in this study, Table S2: Primers used for real-time quantitative PCR.
