# Supplementary material for: Enhanced Expression of Thaumatin-like Protein Gene (LeTLP1) Endows Resistance to Trichoderma atroviride in Lentinula edodes
Source: Life (Basel). 2021 Aug 23;11(8):863. doi: 10.3390/life11080863 (PMC8398078; doi:10.3390/life11080863)
Supplement: Supplementary file 1 [file life-11-00863-s001.zip › life-1323230-supplementary.pdf]

Supplementary Materials

Enhanced Expression of Thaumatin-like Protein Gene (*LeTLP1*) Endows Resistance to *Trichoderma atroviride* in *Lentinula edodes*

Xiaolong Ma[1,2], Xiaolin Fan[1,2], Gangzheng Wang[1,2], Ruiping Xu[1,2], Lianlian Yan[1,2], Yan Zhou[1,2], Yuhua Gong[1,2], Yang Xiao[1,2], Yinbing Bian[1,2,*]

1 Institute of Applied Mycology, College of Plant Science and Technology, Huazhong Agricultural University, Wuhan 430070, China;
2 Key Laboratory of Agro-Microbial Resource and Development, Ministry of Agriculture, Wuhan 430070, China;
* Correspondence: bianyinbinghzaucn@yahoo.com (Y.b.B);

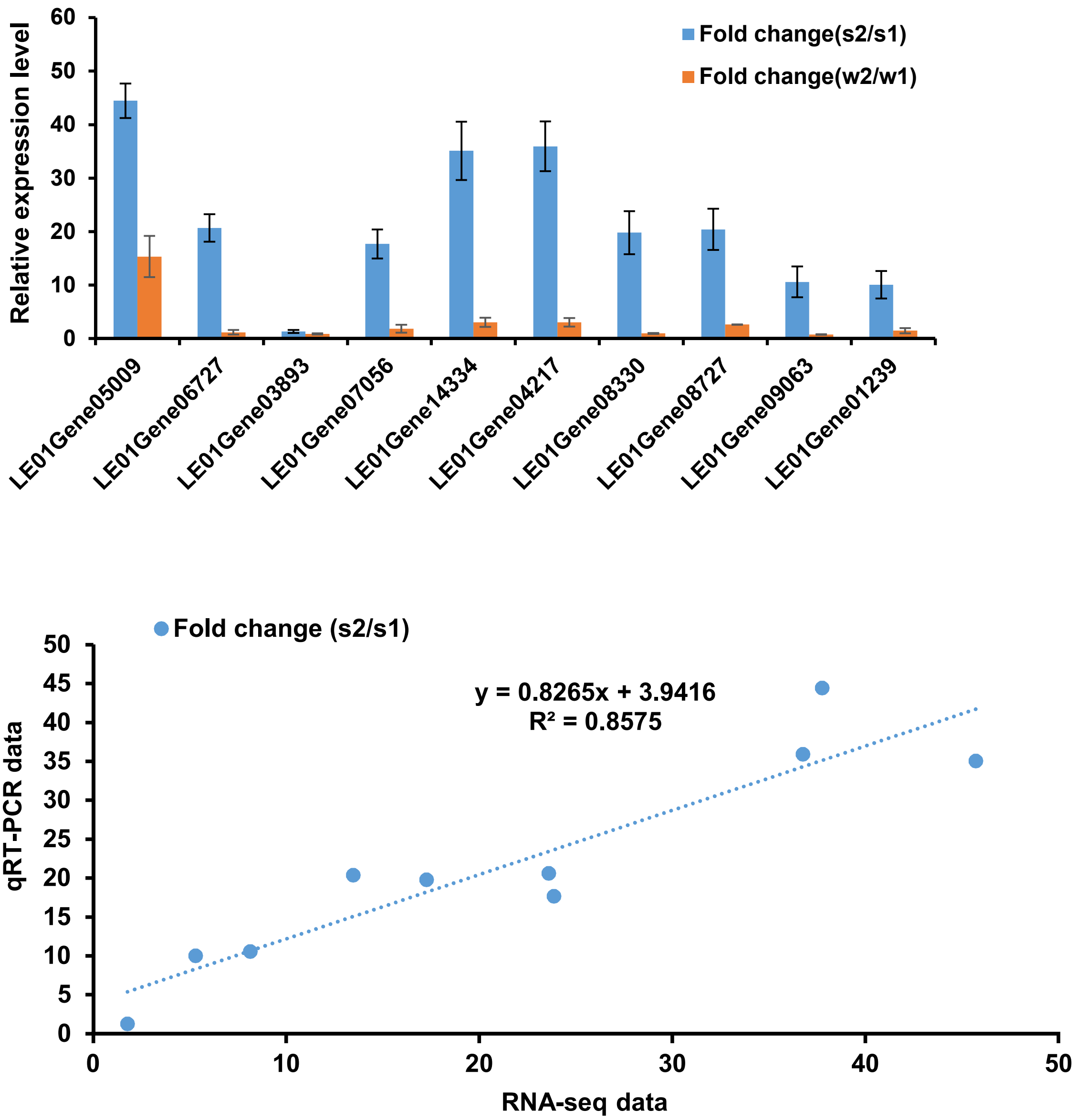

Figure S1. qRT-PCR results of ten selected genes and correlation between transcripotome data and real time PCR results. The resistant and susceptible *L. edodes* mycelia samples (s2, w2) were harvested after the *T. atrovide* strain 92-1 had contact with the *L. edodes* strains of 24 hours, respectively. As control, the Y3334 strain and Y55 strain of *L. edodes* was confronted with itself, respectively. The mycelia samples of resistant and susceptible *L. edodes* (s1, w1) were harvested after contact for 24 hours, respectively. s1 represents Y3334 vs. Y3334; s2 represents Y3334 vs. *T. atrovide* strain 92-1; w1 represents Y55 vs. Y55; w2 reprsents Y55 vs. *T. atrovide* strain 92-1.

```
                               *
LeTLP1        ---MMKNSIIISALFSTFVAVSARTFTVKNNCKYTVWPAIFTDMNVGTDKPSMPTGWEAA    57
LeTLP (Y55)   ---MMKNSIIISALFSTFVAVSARTFTVKNNCKYTVWPAIFTDMNVGTDKPSMPTGWEAA    57
L. edodes     MKFTTAASLVSVGLV--STVDAARTFTVYNGCPFTIWPAMFTGVNQATASPAYTTGWAAN    58

                                       *         *   *    *
LeTLP1        PGNTSTFTVPDDWQSGRIWGRTDCDFSK-PNASSCATGGCNGGLLCDEHSGTGVPPVTVA    116
LeTLP (Y55)   PGNTSTFTVPDDWQSGRIWGRTDCDFSK-PNASSCATGGCNGGLLCDEHSGTGVPPVTVA    116
L. edodes     AYTAVSFSVPDNWQAGRIWARRDCDFSVNPGPNSCLDGGCNGGLVCDPTTGTGVPPASLA    118

              +            +   +            *   *      *
LeTLP1        EWTLGKNGSPDNYDVSMVDGFNIPMSVVPTAGCQAIDCNTDLNPNCPAQLQL-KDASGTV    175
LeTLP (Y55)   EWTLGKNGSPDNYDVSMVDGFNIPMSVVPTAGCQAVDCNTDLNPNCPAQLQL-KDASGTV    175
L. edodes     EFTLSGAGGLDYFDISFVDGYNLPISITNNVNCPAPICAVDLGPNCPAPLAGPFDSTGFP    178

                *   *            **        *             *
LeTLP1        VGCKSACDANLDGDPQNSPNCCSGSHNVPATCPPSGVQFYDYFKNGCKNAYAYAYDESSK    235
LeTLP (Y55)   VGCKSACDANLDGDPQNSPNCCSGSHNVPATCPPSGVQFYDYFKNGCKNAYAYAYDESSK    235
L. edodes     VGCKSACDANLDGDQQNSPNCCSGQYDTPATCPVSGVEYYTYFKDACPDAYAYAYDESSE    238

                   *            *
LeTLP1        TALWTCDSTQNADYAVTFCP--    255
LeTLP (Y55)   TALWTCDSTKNADYAVTFCP--    255
L. edodes     SALWTCADSLNADYTITFCPPA    260
```

Figure S2. Alignment of the amino acid sequences of *Le*TLP1 (Y3334), *Le*TLP (Y55), and *L. edodes* TLP(GenBank: AB244759). Dashes in the sequence represent the introduction of a single amino acid gap. Conserved Cys are indicated with an asterisk, and conserved acidic amino acids in the acidic cleft are indicated with a +.

Table S1 Cultivated and wild strains of *Lentinula edodes* used in this study.

Strain number	Strain name	Origin	Main agronomic traits
c2	S605	Shanghai	S,F,M and MLa
c6	Cr04	Sanming-1	S, F, MH and Ea
c9	L12	Sanming	W, D, ML
c10	L135	Sanming	S, F or D, L and La
c20	L856	Sanming	S, F or D, ML and Ea
c23	S602	Shanghai	S, F, M and La
c27	Guangxiang-51	Guangdong	W, D, ML
c28	Hunong-1	Shanghai	W, D, M
c31	Huaxiang-8	Wuhan	S, F, M and Ea
c42	Qingke-20	Qingyuan	S, D, ML and MLa
c47	Qiu-6	Wuhan	S, D, M and Ea
c48	Rifeng-34	Henan	W, D, L
c49	Senyuan-10	Yichang	W or S, D, L and MLa
c50	Senyuan-1	Yichang	W, D, L
c51	Senyuan-2	Yichang	W or S, D, L and La
c64	430	Wuhan	S, D, ML and Ea
c67	908	Henan	S, D, ML and MLa
c82	868	Zhejiang	S, F, ML and Ea
c85	Xiangjiu	Guangdong	W, D, M
c87	Yuhua-2	Biyang	S, D, ML and Ea
c88	Yuhua-4	Biyang	S, D, ML and Ea
Y1	EFISAAS0229	Jingdong	-
Y5	EFISAAS0351	Jingdong	-
Y7	00167	Yaoan	-
Y8	00168	Xianggelila	-
Y11	ACCC50786	Huangshan	-
Y14	GAN059	Kang couty	-
Y29	HUB039	Shennongjia	-
Y30	HUB040	Shennongjia	-
Y37	HUB091	Changyang	-
Y39	HN002	Chenzhou	-
Y51	SHX044	Liuba	-
Y55	NO.41	Xiushui	-
Y70	LeQc743s	Qingchuan	-
Y76	LeWs735	Dechang	-
Y78	LHLy14	Huili	-
Y79	LHLy217	Huili	-
Y84	LMLH14	Mianning	-
Y88	LMLH36	Mianning	-
Y89	LMLH52	Mianning	-

Y91	LMLH59	Mianning	-
Y94	LMLH116	Mianning	-
Y100	LMLHA18	Mianning	-
Y104	LMLHA36	Mianning	-
Y110	LMLHL22	Mianning	-
Y111	LMLHL26	Mianning	-
Y113	LMLHL210	Mianning	-
Y118	LMYP62	Miyi	-
Y121	LPG82	Puge	-
Y234	YAASM234	Nanhua	-
Y358	YAASM358	Chuxiong	-
Y366	YAASM366	Chuxiong	-
Y1515	YAASM1515	Yangbi	-
Y1518	YAASM1518	Yangbi	-
Y3334	YAASM3334	Longling	-
Y3353	YAASM3353	Jingdong	-

S for sawdust cultivation; W for woodlog cultivation; W or S both for sawdust and wood log cultivations; F (fresh fruiting body production type),fruiting body mostly sold in fresh form in the market; D(dried fruiting body production type),fruiting body mostly sold in dried form in the market; For D, fruiting body sold both in fresh and dried forms in the market; H(high temperature type), fruiting body formation at 8–32°C; MH (middle-high temperature type), fruiting body formation at 15–28°C; M (medium-temperature type), fruiting body formation at 10–25°C; ML (middle-low temperature type), fruiting body formation at 8–23°C; L (low temperature type), fruiting body formation at 5–20°C; Ea (early maturing type), duration from inoculation to fruiting body formation is 65–75 days; MLa (middle-late maturing type), duration from inoculation to fruiting body formation is 90–150 days; La (late maturing type), duration from inoculation to fruiting body formation is 160–200 days . . .

Table S2 Primers used for real time quantitative PCR.

**Gene numbers**	**Gene name**	**Forward primer sequence(5'-3')**	**Reverse primer sequence（5'-3')**
LE01Gene01050.t2	Actin-1	ACCACCGCTGAGC GTGAAAT	CAAGGAAGGCA GGCTGGAAG
LE01Gene05009	Thaumatin-like protein	TGACTTCTCGAAG CCCAACG	TTCCGAGTGTCC ATTCAGCAAC
LE01Gene06727	Oligopeptide transporter 3	GAGAAACCACCTC GCACAGAA	ATCCAAAGCAGC TTCCGACT
LE01Gene03893	Spore germination protein	GTATGGGCAATGG CGTTCTG	TGAGCAGCGATG GTTTGACA
LE01Gene07056	lcc-3	CGACACTGATTAA CGGGAACG	CGATGGAGAAG CCGTGGAA
LE01Gene14334	Cytochrome b561 and DOMON domain-containing protein	GCGGGTGCGGATT ATGTTT	TTGAAAGCCGTC GCCTCTAA
LE01Gene04217	Probable peptidoglycan endopeptidase	AATGAGATCGCCA CCAAGAAC	GTGCCAGGGATG CAAAGCT
LE01Gene08330	lcc-1	ACGGGTAACAAA GGCGACAA	CAAGGGTCCAC GCAATCCA
LE01Gene08727	Oligopeptide transporter 6	TTCGATAGCCCT CGCCTCAT	AGCGCCAAAG CAAGATAGACC
LE01Gene09063	Oligopeptide transporter 2	GTCTTGGGAAAG GTAGCGAGG	CGAGCTCCCGA ACTATCTGTG
LE01Gene01239	Oligopeptide transporter 3	GCTCAATCCTTG GACATTTCGT	GGCGTCCATTC CCATACCAA
